# Optical Coherence Tomography in Huntington's Disease—A Potential Future Biomarker for Neurodegeneration?

**DOI:** 10.3390/neurolint17010013

**Published:** 2025-01-20

**Authors:** Clancy Cerejo, Nicolas De Cleene, Elias Mandler, Katarina Schwarzová, Samuel Labrecque, Philipp Mahlknecht, Florian Krismer, Atbin Djamshidian, Klaus Seppi, Beatrice Heim

**Affiliations:** 1 Department of Neurology, Medical University of Innsbruck, Innsbruck 6020, Austria; 2 Department of Neurology, Hospital Kufstein, Kufstein 6330, Austria

**Keywords:** Huntington's disease, optical coherence tomography, neurodegeneration

## Abstract

Huntington's disease (HD) is a progressive neurodegenerative disorder for which, until now, only symptomatic treatment has been available. Lately, there have been multiple ongoing clinical trials targeting therapeutic agents for preventing disease onset or slowing disease progression in HD. These studies are in constant need of reliable biomarkers for neurodegeneration in HD. In recent years, retinal biomarkers have attracted significant attention in neurodegenerative disorders. Likewise, optical coherence tomography (OCT) is being evaluated as a potential biomarker in HD. In this article, we review the existing literature on OCT as a biomarker for neurodegeneration in HD.

## 1. Introduction

HD is an autosomal dominant progressive neurodegenerative disorder characterized by motor, cognitive, and psychiatric disturbances [1]. It is caused by trinucleotide CAG repeats in exon 1 of the huntingtin (HTT) gene encoding the HTT protein on chromosome 4 [2,3]. The Unified Huntington's Disease Rating Scale is commonly used to provide a standardized assessment of the clinical features and disease progression in HD patients [4,5].

Recently, the Huntington's Disease Integrated Staging System (HD-ISS) has been developed, which defines the various stages of HD, primarily for utilization in clinical research [6]. The HD-ISS staging is mainly based on the biological, clinical, and functional assessments of the HD patients. Accordingly, HD-ISS Stage 0 includes individuals with pathogenic CAG expansion but without any detectable change in pathological biomarkers, signs or symptoms, or any functional changes due to HD. HD-ISS Stage 1 includes individuals with HD-related pathophysiological changes which can be detected by the measurable change in biomarkers, HD-ISS Stage 2 represents the onset of HD-related phenotypical changes (signs and symptoms), and HD-ISS Stage 3 represents the onset of functional decline due to HD. Certainly, the primary benefit of the HD-ISS lies in its potential to reliably categorize and monitor individuals in early disease stages. Implementing the HD-ISS could help streamline the development of clinical trials aimed especially at presymptomatic or prodromal populations, while also allowing for standardized data across current and future studies [6].

With the existing challenges in HD, there is a constant need for sensitive, reproducible biomarkers that can identify changes, especially in the preclinical stage of HD. Over the past years, imaging biomarkers such as volumetric assessments, as well as neurocognitive changes, have been developed for early detection of the preclinical stage of HD [7,8,9]. However, most of these parameters are not sensitive enough and are time-consuming for routine clinical use [10].

In recent years, retinal biomarkers have gained importance in neurodegenerative disorders. The retina is derived from the neuroectoderm which has histological and functional similarities with the neurons in the brain [11,12]. The existing literature on visual dysfunction in HD patients has reported impairments in the structural and functional integrity of the visual pathways in these patients [13]. These retinal structural changes in HD patients can be investigated using optical coherence tomography (OCT) [14,15].

## 2. Optical Coherence Tomography

**Figure 1:**
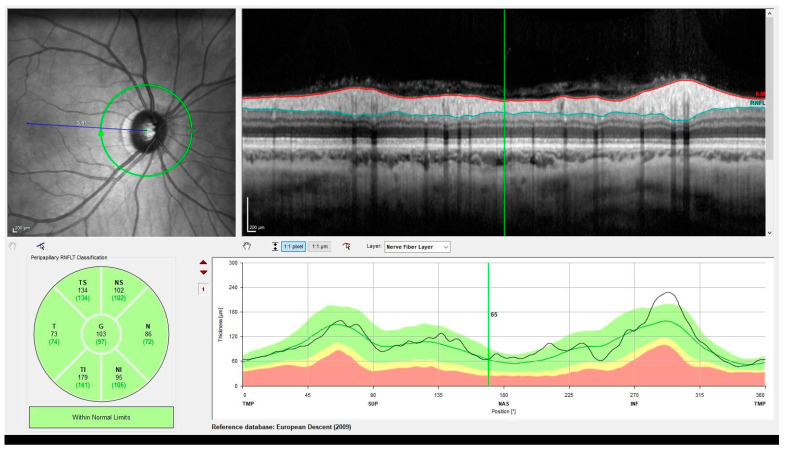
Heidelberg Spectralis optical coherence tomography of normal retinal nerve fibre layer (RNFL) thickness.

OCT is a non-invasive, rapid, quantitative, and user-friendly imaging technique to examine the retina and the different retinal layers including the retinal nerve fibre layer (RNFL) and macula (Figure 1) [16,17]. OCT works on the principle of low-coherence interferometry; in which light is reflected from different retinal layers, and high-resolution, cross-sectional images of the retina are captured. During OCT imaging, light is split into two paths, of which one is directed at the retina and the other at a reference mirror. The light reflected from the retina returns to the interferometer and is compared to the light reflected from the reference arm. An interference pattern is formed based on which detailed images of different retinal layers at a micrometre-scale resolution are generated. This allows for precise detection of subtle changes in retinal layers reflecting neurodegeneration [16,18].

Since its first description, it has been increasingly used to evaluate retinal changes associated with various neurodegenerative disorders such as Alzheimer's disease, Parkinson's disease, or multiple sclerosis [19,20]. The high-resolution capability of OCT has improved over time. These advanced spectral-domain OCT devices are able to accurately image the retinal anatomy [21]. However, there are certain limitations with the use of OCT devices. These include various manufactured models and generations of OCT devices which can influence the scan pattern and the outcome measures [18,22,23,24]. Image quality, resolution, and processing algorithms vary between OCT device models and generations. Devices with lower resolution result in less detailed images of retinal layers and may fail to detect subtle changes in the retina, especially in the early stages of neurodegenerative diseases. In addition, depending on the device software, there may be discrepancies in quantitative assessments such as retinal layer thickness or volume, which may introduce systematic bias, further complicating the comparison of study results. Hence, it is crucial to consider the specific OCT system and scan parameters utilized when comparing findings across different studies.

This review article evaluates the current literature regarding the use of OCT in HD and provides a comprehensive overview of various OCT studies in HD, by bringing together the existing data, exploring the underlying mechanisms contributing to OCT changes in HD, and identifying gaps in the current literature that require further investigation.

Methodologically, we conducted a comprehensive search of the published literature in the Google Scholar and PubMed databases using key terms such as “Huntington's disease” AND “optical coherence tomography” AND “Huntington's disease” AND “retina”. We also screened the cross-references from these articles. We included studies on both premanifest and/or manifest HD patients. Original articles in English were included in the review.

## 3. OCT Findings in Huntington's Disease

In Huntington's disease, OCT is used to measure the thickness of retinal layers such as the retinal nerve fibre layer (RNFL), ganglion cell layer (GCL), and macula. It can detect retinal changes even before the appearance of overt clinical signs. These retinal changes can be correlated with clinical scores or cognitive and motor impairments, which could provide valuable insight into retinal degeneration, particularly in therapeutic HD clinical trials, where it may be used as a potential outcome measure.

In the current review, we found thirteen studies on HD patients reporting OCT findings and comparing them with healthy controls (HCs). Of these, seven studies reported OCT findings in manifest HD patients, two studies reported findings in premanifest HD patients, and the remaining four studies reported OCT data in both premanifest and manifest HD patients. Unified Huntington's Disease Rating Scale—Total Motor Score (UHDRS-TMS) was used to measure the disease severity in all the studies. Out of these thirteen studies, eight studies used Heidelberg Spectralis (Heidelberg Engineering, Heidelberg, Germany) for OCT measurements. Detailed findings from each study are presented in a tabulated format (Table 1) [25,26,27,28,29,30,31,32,33,34,35,36,37], with some of the important parameters discussed below.

Mean total RNFL thickness was reduced in HD (including premanifest and manifest) patients in a study by Svetozarskiy et al. [30] while other studies [25,26,28] showed no such significant decrease. Dusek et al. reported similar results but with a small effect size and the findings did not pass the false discovery rate adjustment [35]. Similarly, in presymptomatic HD patients, a significant decrease in the total RNFL thickness and RNFL thinning in specific quadrants (superior, inferior, and temporal) was reported by Iwona Mazur-Michałek et al. [34].

Temporal RNFL thickness was found to be reduced in HD patients by Kersten et al. [26], Gatto et al. [28], and Svetozarskiy et al. [30] but no difference was reported by Dusek et al. [35]. Additionally, Dusek et al. observed that there was a significant effect of age on temporal RNFL measurements in the intergroup model and proposed that age may be the underlying factor for temporal RNFL atrophy, rather than the disease per se [35].

While comparing average peripapillary RNFL reduction between HD and HCs, Kersten et al. [26], Andrade et al. [27], and Sevim et al. [29] found no significant difference between the groups. However, when comparing temporal peripapillary RNFL thickness, Sevim et al. [29] reported significant differences between HD and HCs.

Regarding total macular volume (TMV), a significant decrease in HD patients was reported by Haider et al. [25] while the others [26,27] reported no significant difference. In a more recent study, Pavel Dusek et al. have reported that the changes in TMV are possibly driven by ageing only and not by changes associated with HD. [35] Similarly, Amini E et al. [32] showed an inverse correlation between macular retinal thickness and the disease duration and age in HD patients.

Ganglion cell complex thickness was studied by Svetozarskiy et al. and was found to be significantly reduced in both premanifest and manifest HD patients as compared to the control group [30]. In contrast, a study by Di Maio et al. [31] on manifest HD subjects and another study by Mazur-Michałek et al. [34] on premanifest HD subjects showed no significant differences in the mean ganglion cell complex thickness as compared to the control group.

## 4. Discussion

Over the past few decades, there has been rising evidence about retinal involvement in neurodegenerative diseases. Multiple studies in humans and animal models have highlighted retinal involvement in HD patients. One of the first reported evidence of retinal dysfunction in HD is the impairment of the retinal increment threshold in HD patients [38]. Subsequent evidence of retinal dysfunction comes from the studies on drosophila and mice HD models [39,40,41]. A study on the R6/1 mice model has shown early progressive retinal degeneration with prominent initial involvement of cones. This conal dysfunction is hypothesized to be caused by the loss of certain proteins involved in the cone phototransduction. Also, with disease progression, there is gradual involvement of rod photoreceptors along with retinal remodelling, gliosis, and cell death [42]. Further, mutant huntingtin aggregates are reported to be found in nuclear layers of R6/2 transgenic mice and appear to selectively affect retinal interneurons [41,43].

However, while these animal studies show evidence of retinal involvement, a single post-mortem study in an advanced HD patient has shown no evidence of macroscopic or histological signs of neurodegeneration in the HD eyes as compared to controls [44].

OCT is increasingly being used to evaluate in vivo retinal changes reflecting ongoing neurodegenerative changes. As described above, OCT findings in HD studies are variable and conflicting. In one of the early studies, Kersten et al. observed that temporal RNFL in HD is preferentially affected and that temporal RNFL thinning and macular volume reduction are significantly correlated with the disease duration. These findings were interpreted with the hypothesis that as the disease progresses, there is atrophy of the small retinal ganglion cells in the macula, and the axons that arise from these macular cells to form the papillo-macular bundle, which extends to the temporal aspect of the optic nerve, may either be directly affected by the disease or are atrophied as a consequence of damage to their cell bodies in the macula [26]. Further, their findings of thinned temporal RNFL are similar to other inherited neurodegenerative disorders with known mitochondrial dysfunction like Friedreich's ataxia and Leber's hereditary optic neuropathy, thereby supporting the implication of mitochondrial dysfunction in HD [45,46]. Next, Andrade et al., in a small cohort of eight patients, showed reduced macular choroid thickness in HD with no difference in average pRNFL thickness or any retinal quadrant. The macular choroid thickness was not related to the disease severity; this was thought to indicate that the observed choroidal changes precede the retinal changes. Based on these findings, the macular choroid was proposed to be a potential structure for the detection of presymptomatic patients, and the macular retina was proposed for monitoring disease progression [27].

Next, Gatto et al. found a significant thinning in temporal and superior sectors of the RNFL thickness in HD patients as compared to the controls. Their results supported some of the findings of Kersten et al. and underpinned the hypothesis of the temporal sector being one of the most frequently affected regions in mitochondrial disorders, which could reflect the possible mitochondrial impairment in HD as well [28].

A step ahead of previous pilot studies, Sevim et al. first analyzed the single retinal layer using OCT in HD. They reported significant thinning in temporal pRNFL in HD; however, no alterations were found in average pRNFL in HD. Further, all the inner retinal layers were thinner in HD which also had significant inverse correlations with disease progression scores, mainly with CAG repeats, disease burden score, and TMS UHDRS. Of these parameters, the ganglion cell layer (GCL) was reported to be the most significant retinal biomarker for disease progression [29]. Their findings were similar to previous studies on other neurodegenerative disorders like multiple sclerosis, which reported that the GCL and inner plexiform layer (IPL) were more frequently altered and that the ganglion cell inner plexiform layer complex thickness had better sensitivity than temporal pRNFL thickness for detecting changes in retinal thickness and predicting axonal damage [47,48]. Similar findings were reasoned out to be because of the probable affection of neuronal cell bodies earlier than the retinal axons in multiple sclerosis [49].

Svetozarskiy et al. studied OCT parameters in premanifest and manifest HD patients. They reported significant thinning of the subfoveal choroid, mean ganglion cell complex (GCC), mean RNFL thickness, and temporal RNFL thickness in premanifest and manifest HD subjects thereby concluding that macular choroid atrophy, ganglion cells, and axonal degeneration start early in the course of the disease [30]. The macular choroid thinning in HD was explained to be a consequence of intracranial hemodynamic disturbances, probably associated with abnormal huntingtin accumulation in the cerebral vessels as described by Drouin-Ouellet et al. [50]. They also proposed that the ganglion cells and RNFL thinning have a specific pattern of preferential parvocellular degeneration, which is probably associated with mitochondrial dysfunction [30,51].

In a pilot study, Di Maio et al. explored the potential of optical coherence tomography angiography (OCTA) to examine retinal and choriocapillaris vascular networks in HD. While their findings did not reveal any significant changes in vessel density across different macular regions, the study offered valuable insights into the use of OCTA in HD. Additionally, in contrast to the previous studies, their study demonstrated no statistically significant differences in GCC and RNFL parameters between HD patients and controls [31].

Two studies by Schmid et al. and Mazur-Michałek et al. carried out OCT measurements, particularly on presymptomatic HD patients, and compared them to healthy controls. Schmid et al. reported that the OCT parameters (IR thicknesses of the GCL, ganglion cell inner plexiform layer (GCIPL), and total retina) were not robustly different between pre-HD and HCs [33]. They argued the finding could be because their pre-HD cohort was more than 16 years before disease onset. In addition, they reported a marginal but significantly higher RNFL in pre-HD which was considered to be a counterintuitive result, possibly a result of centre bias or a small HCs cohort with a broader age range. Overall, they proposed that OCT may not be sensitive enough to detect early changes [33]. Mazur-Michałek et al. also reported no differences in GCC parameters as compared to the HCs [34]. However, contrary to Schmid et al., they reported a significant reduction in total RNFL thickness including specific quadrants like superior, inferior, and temporal. These findings were partly similar to the results of Gatto et al. and Kersten et al. [26,28].

Murueta Goyena et al. first studied the relationship between retinal parameters and cognition in premanifest and manifest HD. They reported a significant association between the macular thickness and MoCA scores, with the inner nuclear layer showing the largest regression coefficient, thereby proposing OCT to be a potential biomarker of cognitive status in manifest HD [36].

More recently, Joseph et al. studied the foveal avascular zone (FAZ) area, a new OCT parameter, and reported it to be decreased in the HD group compared to controls. The FAZ area is situated over the foveal pit, which is composed exclusively of photoreceptors, specifically the cones [52]. Based on this, the changes in HD were hypothesized to be due to possible disease-associated photoreceptor degeneration already reported in mice models [39,41,42]. Previous studies on other neurodegenerative disorders have shown mixed results with regard to the FAZ area changes [53,54]. Furthermore, no other studies on HD have evaluated the FAZ area; hence, the clinical significance of this finding as of now remains uncertain [37].

Finally, a recent meta-analysis of OCT studies showed significant thinning of the average and temporal pRNFL and subfoveal choroid in manifest HD as compared to HCs. However, the premanifest HD group showed no significant differences when compared to the HCs. The results from this study offer compelling evidence that OCT metrics have the potential to serve as a biomarker for the assessment of retinal changes associated with HD [55].

## 5. Conclusions

As discussed above, retinal characteristics have garnered significant attention as a potential biomarker in early diagnosis and monitoring progression of the neurodegenerative process in HD patients. However, the results from existing studies show conflicting results, which could be due to multiple reasons like small sample sizes or different age groups of the study population. It could also be because of patients being in different disease stages in different study cohorts. Using a unified staging system like the HD-ISS to classify HD patients in future research studies may prove helpful in resolving discrepancies especially when comparing data across various studies. In addition, because OCT requires patient cooperation, with the disease progression, chorea and cognitive impairments worsen, thereby limiting the usefulness of the OCT measurements. For studies involving premanifest HD patients, the estimated age until disease onset could also influence the study results. Also, all the existing studies are cross-sectional, and longitudinal observational studies with larger sample sizes seem necessary to resolve the disparity among these study results. Further, limited data exist on retinal parameters, particularly on single retinal layers with HD pathology. More studies targeting these single retinal layer parameters like the ganglion cell layer, nuclear layer, and plexiform layer could provide further insight into retinal biomarkers in HD.

Lastly, artificial intelligence, especially machine learning, is being increasingly incorporated into the field of neurosciences. A recent paper on Alzheimer's disease biomarkers, combining OCT measurements with machine learning, showed that the machine learning algorithms were superior to the traditional logistic regression in their diagnostic performance [56]. Similarly, applying such machine learning algorithms with OCT measurements in HD could boost the diagnostic and prognostic potential of retinal biomarkers to detect neurodegeneration in HD.

## Figures and Tables

**Table 1:** Review of the literature.

Study	Device	No. of Patients/HD Eyes	Main Results (Comparing the HD and/or Pre-HD Groups with Healthy Controls (HCs))
1. Haider S et al. (2014) [25]		51 (HCs; premanifest, early, moderate, juvenile stage HD)	Significantly reduced in HD: Macular volume No significant thinning in HD: RNFL
2. Kersten HM et al. (2015) [26]	Heidelberg Spectralis (Heidelberg Engineering, Germany)	20 manifest eyes, 6 premanifest eyes, 29 control eyes	Significantly reduced in HD: Temporal RNFL thickness No significant difference between the HD and controls: Average peripapillary RNFL thickness Macular volume Macular thickness No significant difference between the premanifest HD and manifest HD: Average RNFL thickness Temporal RNFL thickness Macular volume
3. Andrade C et al. (2016) [27]	Spectralis HRA + OCT, Heidelberg Engineering, Germany	15 eyes of 8 HD patients, 16 eyes of 8 HCs	Significantly reduced in HD: Macular choroidal thickness (average, centre, and inferior) No significant difference between the HD and controls: Macular retinal thickness Peripapillary RNFL thickness (average and different RNFL quadrants) Peripapillary choroidal thickness
4. Gatto E et al. (2018) [28]	Heidelberg Spectralis OCT plus (Heidelberg Engineering, Inc, Germany)	27 eyes of 14 HD patients, 26 eyes of 13 HCs	Significant thinning in HD: Temporal and superior RNFL thickness No significant difference between the HD and controls: Mean total RNFL thickness Inferior and nasal RNFL thickness
5. Gulmez Sevim D et al. (2019) [29]	Heidelberg Engineering, Germany	15 eyes of 15 HD patients, 15 eyes of 15 HCs	Significant decrease in the thicknesses of: Temporal, superotemporal, and inferotemporal pRNFL Macular RNFL Ganglion cell layer Inner and outer plexiform layer Inner nuclear layer Outer plexiform layer Significant increase in the thicknesses of: Outer nuclear layer Outer retinal layer Significant decrease in the volumes of: Retinal pigment epithelium layer Inner plexiform layer Outer macular layer No significant difference in the HD and control groups in: Average pRNFL thicknesses Central foveal thickness
6. Svetozarskiy S et al. (2020) [30]	RTVue-100 spectral-domain OCT; Optovue Inc., Fremont, CA, USA	29 premanifest eyes, 31 manifest eyes, 31 HCs eyes	Significantly reduced thickness in HD (premanifest and manifest) Subfoveal choroid Mean Ganglion cell complex Mean RNFL thickness Temporal RNFL thickness Significantly reduced thickness in manifest HD: Nasal and inferior RNFL thickness
7. Di Maio LG et al. (2021) [31]	Software RTVue XR version 2017.1.0.151, Optovue Inc., Fremont, CA, USA)	32 eyes of 16 HD patients, 26 eyes of 13 HCs	Significantly reduced in HD: Central choroidal thickness No significant differences between HD and control groups: Ganglion cell complex parameters RNFL parameters Retinal and choriocapillaris vessel density
8. Amini E et al. (2022) [32]	Heidelberg Engineering, Germany	46 eyes of 25 HD patients, 50 eyes of 25 HCs	Significantly reduced in HD: Macular thickness (inner and outer superior sectors and inferior–outer sector) Inferior pRNFL thickness No significant differences between HD and control groups: Superficial and deep plexus capillary density
9. Schmid RD et al. (2022) [33]	Spectralis, HRA + OCT, Heidelberg Engineering	24 presymptomatic HD patients, 38 HCs	RNFL inner ring (IR) thickness was significantly higher in pre-HD participants No significant difference between pre-HD and HCs in the IR thicknesses of the ganglion cell layer, ganglion cell+ inner plexiform layer thickness, and total retina
10. Mazur-Michałek I et al. (2022) [34]	Revo NX 110 (Optopol, Zawiercie, Poland) spectral domain OCT	13 presymptomatic HD patients, 14 HCs	Significant decrease in presymptomatic HD: Total RNFL thicknesses including specific quadrants: superior, inferior, and temporal No significant difference in the pre-HD and control groups: Dimensions of the optic nerve head cup and disc Macular retinal thinning Average ganglion cell complex thickness Average retinal thickness
11. Dusek P et al. (2023) [35]	Heidelberg Spectralis	94 eyes of 41 HD patients *, 82 eyes of 41 HCs	Significant difference in the HD and control group (not passing false discovery rate adjustment and with small effect size): Global mean RNFL thicknesses Total macular volume No significant difference in the HD and control groups in: Temporal RNFL thickness
12.Murueta-Goyena A et al. (2023) [36]	Spectralis, Heidelberg Engineering	32 eyes of 16 premanifest HD carriers, 38 eyes of 20 manifest HD patients, 72 eyes of 36 HCs	Premanifest HD and manifest HD patients had thinner retinal external limiting membrane-Bruch's membrane complex. Manifest HD had a thinner temporal peripapillary retinal nerve fibre layer compared to controls No significant difference between premanifest HD and manifest HD and HCs in total macular thickness
13. Joseph S et al. (2024) [37]	Zeiss Cirrus HD-OCT 5000 with AngioPlex OCTA (Carl Zeiss Meditec, Dublin, CA, USA)	44 eyes of 23 HD patients, 77 eyes of 39 HCs	Significantly reduced in HD: Average GCIPL thickness FAZ area

* Four of the patients were measured twice during the study period. Abbreviations: HCs, healthy controls; HD, patients with Huntington's disease; RNFL, retinal nerve fibre layer; pRNFL, peripapillary RNFL; GCL, ganglion cell layer; GCIPL, ganglion cell inner plexiform layer; FAZ, foveal avascular zone.

## Data Availability

Data sharing is not applicable to this article as no datasets were generated or analyzed during this study.
